# Effect of Vestibular-Oriented Balance Training on Postural Control and Risk of Fall in Patients With Parkinson's Disease

**DOI:** 10.1155/nri/6846267

**Published:** 2025-02-25

**Authors:** Mohamed Khallaf, Hatem Jaber, Mansoor Alameri, Dina Magdy, Hend Kamal, Mohamed Hassanin, Mohamed Mousa, Eman Fayed

**Affiliations:** ^1^Department of Physical Therapy, University of St. Augustine for Health Sciences, Austin, Texas, USA; ^2^Department of Physical Therapy for Neuromuscular Disorders and Its Surgery, Faculty of Physical Therapy, Cairo University, Giza, Egypt; ^3^Department of Physical Therapy for Cardiopulmonary Disorders and the Elderly, Faculty of Physical Therapy, Misr University for Science and Technology, Giza, Egypt

**Keywords:** balance training, functional gait, Parkinson's disease, postural control, risk for fall, vestibular

## Abstract

**Background:** Parkinson's disease is a neurodegenerative disorder that affects balance and increases the risk of falling by compromising vestibular signal processing.

**Objectives:** This study aims to assess the impact of vestibular-oriented balance training on postural control and fall risk among people in the middle stages of PD.

**Methods:** Forty middle-stage individuals with PD were assigned to the vestibular-oriented balance training (study group) or the traditional balance training (control group). Outcome measures including Functional Gait Assessment (FGA) and modified Clinical Test of Sensory Interaction on Balance (mCTSIB) using the Biodex Balance System were measured before, immediately after and 4 weeks after treatment.

**Results:** There was a significant group interaction by time for all outcome measures (*p* < 0.001). The results showed that the difference in the FGA and mCTSIB scores from baseline was significant between the two groups at all time points (*p* < 0.001). The study group showed significant sustained improvements in the FGA score overtime, while the control group had a significant improvement at Week 8 but that did not last to Week 12. In mCTSIB, the study group improved significantly in all test conditions (*p* < 0.001), while the control group showed significant improvement only in Conditions 1 and 2, without lasting effects at Week 12 (*p* > 0.05).

**Conclusions:** The findings indicate that the implementation of vestibular-oriented balance training during the middle stage of PD might have a notable and lasting impact on both postural control and the risk of falls.

## 1. Introduction

Parkinson's disease (PD) is a chronic, progressive neurologic illness that manifests with both motor and nonmotor symptoms. Motor symptoms are often the focus of research and treatment of PD [1], but nonmotor symptoms can adversely affect postural control and increase the risk of falling for people with PD [2]. Vestibular system deficits, along with impaired central integration of vestibular information, are identified and evident even in the early stages of the disease process [3–5] and can have a negative effect on postural control and quality of life, and they increase the risk of falling among people with PD. Current evidence revealed that vestibular defects occur more frequently in individuals with PD [6, 7]. This may partially explain the higher incidence of falls observed in individuals with PD compared to controls matched in age [8, 9].

Although the relationship between vestibular functions and PD has been fairly studied [10], little is known about the effect of balance training focused on the vestibular component of postural control on different aspects of gait and the risk of falls among people with mild to moderate PD. Evidence suggests that various external cues, such as visual, auditory, and tactile cues, may be helpful in addressing motor difficulties in PD [11]. However, the long-term advantages of using cues have been doubted [12], as they have not demonstrated any impact in situations without cues [13, 14] or in everyday activities. Additionally, incorporating these cues during early rehabilitation for PD may be a compensatory strategy that hinders the contributions of the vestibular system to postural stability, potentially leading to counterproductive outcomes.

It has been proven that people with PD can benefit from exercise-induced motor relearning, and exercise that may enhance plasticity-related events, including corticomotor excitation, and changes in brain-derived neurotrophic factor levels, especially in the early stages [15, 16]. However, many neurorehabilitation techniques based on neural plasticity principles with a focus on skills or activities that can be generalized or transferred to real-world activities, such as increased independence in the home environment that may decrease the risk of falling, are not yet studied for people with PD.

Conventional balance training for patients with PD typically consisted of static and dynamic components. The specific approaches used to address balance varied between interventions, but many studies incorporated multimodal balance training, which included strengthening exercises, anticipatory postural adjustments, gait training, and functional task practice. However, it is important to exercise caution when interpreting the findings of this research, as the studies that also evaluated fall risk outcomes either favored the control group or did not demonstrate significant differences between the balance intervention and control groups [17].

The impact of balance training on falls in PD is inconclusive. Multiple studies have investigated the relationship between balance training and fall rates, but no significant effects have been observed in some of these studies. However, a particular study that focused on reducing risk factors for falls through a minimally supervised home exercise program over a period of 6 months found a decrease in falls among people with mild PD, while no such reduction was seen in those with more severe PD [18]. Due to conflicting findings, it is currently uncertain which specific components of balance training are necessary to effectively reduce the rate of falls. More research is needed to identify and establish the essential elements of balance training that can provide more informed guidelines for practice.

As a result, the research questions were as follows: (1) would it be helpful to implement vestibular-focused balance training in a rehabilitation program for people with PD? and (2) what is the effect of that on different aspects of gait, postural control, and the risk of falls? The hypothesis of this study was that vestibular-focused balance training may improve dynamic balance and postural control, stability during walking, and movement quality and decreases the risks of falling in PD patients.

## 2. Methods

**Figure 1:**
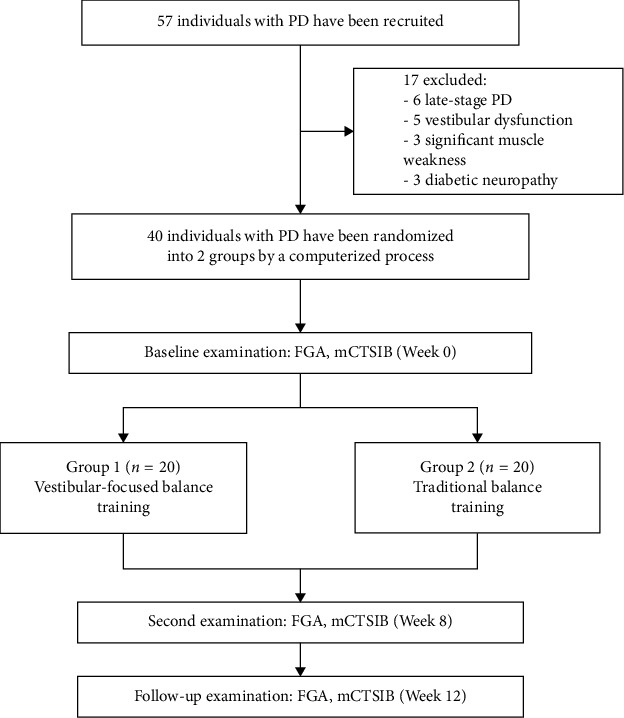
Design and flow of participants through the study. FGA, functional gait assessment; mCTSIB, modified Clinical Test of Sensory Interaction on Balance.

Individuals aged 67 to 84 years with a diagnosis of idiopathic PD were recruited to participate in an 8-week randomized controlled trial. Forty community-dwelling individuals were recruited from the outpatient clinic at the Faculty of Physical Therapy, Cairo University, Egypt. Participants had to be in the middle stage of PD (defined as modified Hoehn and Yahr 2.5 or 3) and under stable PD drug therapy. Additionally, individuals should have a Mini-Mental Status Exam-1 result of more than 23 and a normal sensory state at bedside that includes position and movement sense. Patients are excluded from the study if they have a history of unilateral or bilateral peripheral vestibular disorders, including vestibular neuritis, labyrinthitis, superior canal dehiscence, acoustic neuroma, or Meniere's disease. Patients were also excluded if they have a history of diabetic neuropathy, stroke, multiple sclerosis, or cardiopulmonary disease that could affect their participation in the study. Furthermore, PD patients were excluded if manual muscle testing of the bilateral lower extremity muscles showed significant weakness (less than grade 4/5) or their attendance to exercise was less than 75% (12 out of 16 sessions) or if their PD medications were modified during the study period. The researchers randomly assigned the participants to the study group (G1) and the control group (G2) using a computerized process. Randomization was kept hidden until the last evaluation was complete. The study was approved by the Research Ethics Committee of the Faculty of Physical Therapy of Cairo University (P.T.REC/012/004195 dated 13/11/2022). After receiving verbal and written information on the study procedures to ensure the participant's right to withdraw from the study at any time, all participants gave their signed informed consent to participate in the study. All the study procedures are in accordance with the Declaration of Helsinki of 1964 and its subsequent amendments or comparable ethical standards (Figure 1). The datasets generated and analyzed during the current study are available from the corresponding authors upon reasonable request.

The outcome measures of this study included Functional Gait Assessment (FGA). FGA was used to assess dynamic postural stability during walking, assess an individual's ability to perform multiple motor tasks while walking, and predict/identify the risk for fall within the subsequent 6 months [19]. The FGA has 10 items including gait on level surface, change in gait speed, gait with horizontal and vertical head turns, gait with 180° pivot turn, stepping over obstacles, gait with narrow base of support (NBOS), gait with eyes closed, backward gait, and stairs.

The modified Clinical Test of Sensory Interaction on Balance (mCTSIB) is a standardized clinical test used to assess an individual's ability to maintain balance under various sensory conditions. It evaluates the contribution of different sensory systems (vestibular, visual, and somatosensory) to postural stability. The test is commonly used to assess balance deficits, identify potential causes of balance impairments, and design appropriate rehabilitation strategies [20]. The mCTSIB typically involves the participant standing on a firm surface and maintaining balance in four different sensory conditions: eyes open, firm surface; eyes closed, firm surface; eyes open, foam surface (reducing somatosensory input); and eyes closed, foam surface (reducing both visual and somatosensory inputs). For this study, mCTSIB is being conducted using the Biodex Balance System that combines the principles of balance assessment and sensory interaction with the capabilities of the Biodex system to provide a more advanced, objective, and comprehensive evaluation. The Biodex Balance SD System (Biodex Medical Systems Inc., USA) was used to measure the sway index during the four conditions of the mCTSIB. The mCTSIB protocol of the Biodex Balance System has been well documented in the literature as an effective test to identify individuals with mild-to-severe balance deficits, since it also isolates which system is impaired [20]. Data (FGA and mCTSIB) were collected a few days before starting the interventions, after 8 weeks at the end of the rehabilitation program, and 4 weeks after the end of the treatment programs.

For the interventions, the control group (G2) received traditional flexibility, strength, and balance training. Exercises in the traditional therapy program included static hamstring and calf stretch, leg muscles (calf, anterior tibial, and fibular muscles of the leg) strengthening exercises using resistance bands in all directions, transition from standing on heels to standing on toes with and without upper extremity support, and standing with NBOS followed by half tandem and tandem standing on a stable surface with eyes open and closed.

**Figure 2:**
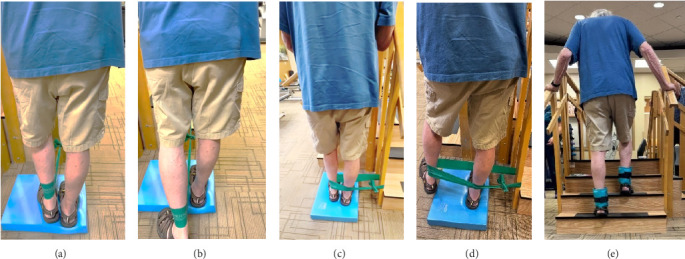
Examples of exercises used to enhance the use of the hip strategies for balance. Extension abduction while standing on a foam pad; included elastic band that resisted hip movement backward (a, b) and to the side (c, d) steps up using a six-inch step with 4 pounds of ankle weight (e).

**Figure 3:**
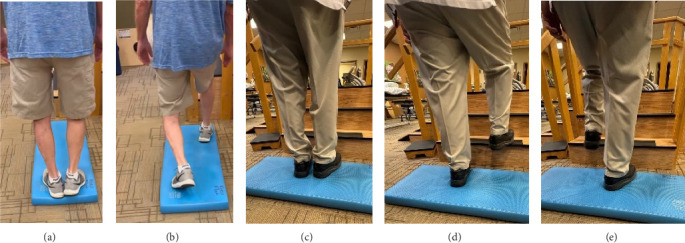
Examples of exercises used for fall prevention. Forward lunges on an extra-long foam pad (a, b) standing on a foam pad and tapping by forefoot on an eight-inch step placed on the front and to both sides (c–e).

**Figure 4:**
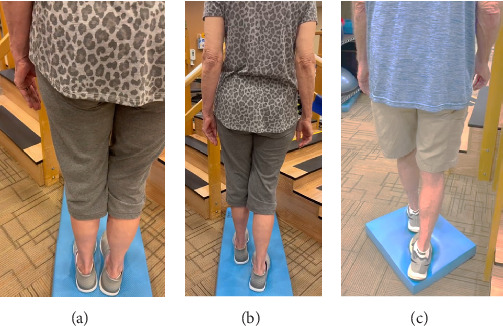
Exercises to promote vestibular input utilization. The base of support (BOS) varied narrow BOS (a), half tandem (b), or tandem standing (c).

In addition to the traditional exercise therapy program applied in the control group, the study group (G1) participants received training that focuses on improving the vestibular component of postural control. The vestibular-focused training included interventions to promote hip strategies for balance, which is mainly controlled by the vestibular system [21, 22] interventions for promoting stepping strategies for fall prevention [23] and strategies to improve utilization of vestibular inputs. Interventions to promote hip strategies for balance (Figure 2) included elastic band that resisted hip abduction, extension, flexion, and adduction while standing on a foam pad; side and forward steps using six-inch step with 4 pounds of ankle weight; lateral waist pull on foam with side, back, and forward steps; and bilateral shoulder extension/flexion using resistance band attached to a wall bar while standing on a foam pad. For fall preventions (Figure 3), the following exercises were applied: forward and side lunges on an extra-long foam pad, step up an eight-inch step and landing on a foam pad, and standing on a foam pad and tapping by forefoot on an eight-inch step placed on the front and to both sides. To promote vestibular input utilization (Figure 4), patients were asked to stand on a foam pad with both eyes engaged with a moving target in front of them followed by head turns to the right/left and up/down, then with eyes closed. Standing on the foam, catching and throwing a ball in different directions. The BOS varied between normal BOS, NBOS (feet together), half tandem, or tandem standing. The patients were also instructed to march in place while standing on the extra-long balance pad. To keep the rhythm of marching and engage the eyes, patients were asked to follow the color-changing light in front of them. Patients also practiced walking 200 feet on a compliant surface while turning their head right/left and then up/down twice, followed by two times of doing the same while counting down by two, starting at 100.

For both G1 and G2, each repetitive exercise was applied in three sets of 12 repetitions. A latex-free green exercise band was used for all exercises that required resistance. Patients were given 1 min of rest between each set of exercises. Other interventions including standing on stable or compliant surfaces or moving on the balance pad were applied for 90 s three times per session with 1 min rest between repetitions. Interventions to improve muscle flexibility were applied in a set of three repetitions with a hold time of 60 s each with 30 s rest between repetitions. The study group patients were instructed to perform identical exercises, consisting of the same sets and repetitions, at home on days when they did not receive therapy. During the initial session, they received detailed instructions and flow sheets. A checklist was also provided to help ensure their adherence to the home exercise program.

### 2.1. Data Analysis

A sample size of 40 subjects was estimated using a moderate effect size for the group × time interaction (partial *η*^2^ = 0.06) [24], alpha level of 0.05, a power of 0.80, and a dropout rate of 40%. The sample size was calculated using the G∗Power software (Version 3.1.2, University of Dusseldorf, Dusseldorf, Germany).

Data were summarized using mean and standard deviation (SD) for quantitative variables and counts (%) for qualitative variables. The normality of continuous variables was examined using the Shapiro–Wilk test and Q–Q normality plots. The distribution of the characteristics of the subjects by study group was evaluated using the chi-square test for qualitative variables and the independent *t*-test for quantitative variables. The median (minimum and maximum) frequency of falls between the two groups was compared using the Mann–Whitney *U*-test.

A 2 × 3 mixed factorial analysis of variance (group x time) was used to examine changes in quantitative outcome variables by study group over time. The analysis included a comparison between groups using the effect of group × time interaction in mixed factorial ANOVA. If the interaction was statistically significant, the change from baseline between the groups was compared at each time point using an independent *t*-test. If the interaction was not statistically significant, the comparison between groups was considered not statistically significant. However, if the main effect of time was significant in mixed factorial ANOVA, a one-way repeated measures ANOVA with post hoc Bonferroni was used to examine changes over time within groups separately. The level of significance was set at *p* ≤ 0.05. Statistical analysis was performed using IBM SPSS software Version 28 for Windows (New York, USA).

## 3. Results

Forty subjects with a mean age of 77.2 ± 4.6 years, a weight of 78.7 ± 6.1 kg, a height of 1.7 ± 0.05 m, and a BMI of 26.8 ± 1.7 kg/m^2^ participated in this study. Eighty-five percent of the subjects were men (*n* = 34). The distribution of all quantitative variables was approximately normal (Field, 2013). There were no significant differences in subjects' characteristics or baseline measures by study group (Table 1). All underlying assumptions to use parametric statistics using (Levene's test for equal variance of errors, Box's test for equality, and Mauchly's test of sphericity) were found valid (Field, 2013). Furthermore, none of the participants reported receiving additional care during the study period. Demographic and general characteristics are presented in Table 1.

### 3.1. Number and Frequency of Falls

**Figure 5:**
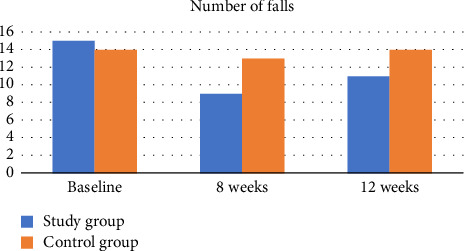
The number of falls in patients presented in the study and the control groups at baseline, 8 weeks, and 12 weeks.

Results showed that the proportion of falls reported by the experimental group during the 8-week intervention period was lower than that reported by the control group. About 45% (*n* = 9) of the experimental group reported at least one fall versus 65% (*n* = 13) for the control group (*χ*^2^ = 1.62, *p* > 0.05; odds ratio = 0.44; 95% confidence interval [0.12 and 1.57]), which indicates that subjects in the experimental group are 55% less likely to report at least one fall compared to controls. However, it is important to note that the difference in fall proportion was not statistically significant (*p* > 0.05), suggesting that the observed reduction may not conclusively represent a true effect of the intervention. The experimental group had a marginal significant drop (30%) in the number of falls by Week 8 compared to only (5%) in the control group (*p*=0.038 without Yates correction; *p*=0.09 with Yates correction) (Table 2). There were no serious fall-related injuries reported by either group. Among those who reported at least one fall, frequency of falls by study group was compared. There was no significant difference in median (minimum, maximum) frequency of falls between the experimental and control groups (*p* > 0.05) (Table 3, Figure 5).

### 3.2. Between-Groups Analysis

**Figure 6:**
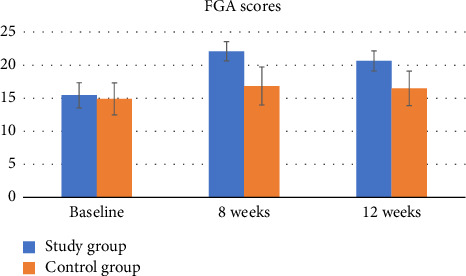
The functional gait assessment (FGA) scores in patients represented the study and the control groups at baseline, 8 weeks, and 12 weeks.

For FGA, there was a significant group by time interaction (*p* < 0.001, *η*^2^ = 0.38). The results of the independent *t*-test showed that the difference in the FGA score from baseline was significant between the two groups at all time points (*p* < 0.001) (Table 4, Figure 6).

For the mCTSIB Condition 1, stable surface open eye condition (SSEO), there was a significant group-by-time interaction (*p* < 0.001, *η*^2^ = 0.21). The results of the independent *t*-test showed that the difference in the mCTSIB SSEO score from baseline was significant between the two groups at all time points (*p* < 0.001) (Table 4).

For the mCTSIB Condition 2, stable surface closed eye condition (SSEC), there was a significant group-by-time interaction (*p* < 0.001, *η*^2^ = 0.29). The results of the independent *t*-test showed that the difference in the mCTSIB SSEC score from baseline was significant between the two groups at all time points (*p* < 0.001) (Table 4).

For the mCTSIB Condition 3, the compliant surface eyes open condition (CSEO), there was a significant group-by-time interaction (*p* < 0.001, *η*^2^ = 0.58). The results of the independent *t*-test showed that the difference in the mCTSIB CSEO score from baseline was significant between the two groups at all time points (*p* < 0.001) (Table 4).

**Figure 7:**
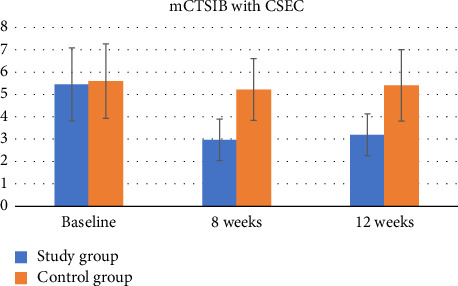
The sway index scores of Condition 4 of the modified Clinical Test of Sensory Interaction in Balance (mCTSIB) which is tested while standing on the compliant surface (foam) with eyes closed (CSEC) in patients represented the study and the control groups at baseline, 8 weeks, and 12 weeks.

For the mCTSIB Condition 4, compliant surface eyes closed condition (CSEC), there was a significant group-by-time interaction (*p* < 0.001, *η*^2^ = 0.60). The results of the independent *t*-test showed that the difference in the mCTSIB CSEC score from baseline was significant between the two groups at all time points (*p* < 0.001) (Table 4, Figure 7).

### 3.3. Within-Group Analysis

For FGA, there was a significant improvement in the study group over time (*p* < 0.001, *η*^2^ = 0.94). The post hoc Bonferroni comparison revealed that the FGA score significantly improved from baseline by Week 8 (*p* < 0.001, Cohen's *d* = 4.80) and Week 12 (*p* < 0.001, Cohen's *d* = 3.62) and then decreased significantly between Week 8 and Week 12 (*p* < 0.001, Cohen's *d* = 1.54), but it remained significant from baseline. Similarly, in the control group, the FGA score improved significantly over time (*p*=0.01, *η*^2^ = 0.21). However, the post hoc Bonferroni comparison revealed that this improvement was only significant between baseline and Week 8 (*p*=0.03, Cohen's *d* = 0.66) (Table 5).

For the mCTSIB SSEO score, there was a significant improvement in the study group over time (*p* < 0.001, *η*^2^ = 0.69). The post hoc Bonferroni comparison revealed that the SSEO score improved significantly from baseline at Week 8 (*p* < 0.001, Cohen's *d* = 1.57) and Week 12 (*p* < 0.001, Cohen's *d* = 1.58), but there was no significant change between Week 8 and Week 12 (*p*=0.18, Cohen's *d* = 0.44). Although the results showed a significant change in the mCTSIB SSEO score over time for the control group (*p*=0.04, *η*^2^ = 0.18), the post hoc comparison revealed no significant change from baseline (*p* > 0.05) (Table 5).

For the mCTSIB SSEC score, there was a significant improvement in the study group over time (*p* < 0.001, *η*^2^ = 0.83). The post hoc Bonferroni comparison revealed that the SSEC score improved significantly from baseline at Week 8 (*p* < 0.001, Cohen's *d* = 2.71) and Week 12 (*p* < 0.001, Cohen's *d* = 2.13), but there was no significant change between Week 8 and Week 12 (*p*=1.00, Cohen's *d* = 0.20). Similarly, in the control group, the mCTSIB SSEC score improved significantly over time (*p*=0.001, *η*^2^ = 0.67). The post hoc Bonferroni comparison revealed that the SSEC score improved significantly from baseline at Week 8 (*p* < 0.001, Cohen's *d* = 1.71), and Week 12 (*p* > 0.001, Cohen's *d* = 1.20) and then significantly lost some of the improvement between Week 8 and Week 12 (*p*=0.015, Cohen's = 0.72) (Table 5).

For the mCTSIB CSEO score, there was a significant improvement in the study group over time (*p* < 0.001, *η*^2^ = 0.87). The post hoc Bonferroni comparison revealed that the CSEO score improved significantly from baseline at Week 8 (*p* < 0.001, Cohen's *d* = 3.06) and Week 12 (*p* < 0.001, Cohen's *d* = 2.51) and then regressed significantly between Week 8 and Week 12 (*p*=0.010, Cohen's *d* = 0.66), but it remained significant from baseline. However, in the control group, there was no significant change over time (*p* > 0.05) (Table 5).

For the mCTSIB CSEC score, there was a significant improvement in the study group over time (*p* < 0.001, *η*^2^ = 0.84). The post hoc Bonferroni comparison revealed that the CSEC score improved significantly from baseline at Week 8 (*p* < 0.001, Cohen's *d* = 2.57) and Week 12 (*p* < 0.001, Cohen's *d* = 2.08), but there was no significant change between Week 8 and Week 12 (*p*=0.40, Cohen's *d* = 0.46). However, in the control group, there was no significant change over time (*p* > 0.05) (Table 5).

## 4. Discussion

The present study aimed to investigate the effects of balance training dedicated to facilitating and strengthening vestibular system inputs on postural control under various sensory conditions, dynamic postural stability during walking, ability to perform multiple motor tasks while walking, and may decrease the risks of fall among individuals suffering from mild-to-moderate PD. The main findings of this study were [1] vestibular-focused balance training significantly improved dynamic postural stability during walking, ability to perform multiple motor tasks while walking, and decreased the risks of fall [2]. Vestibular-focused balance training significantly improved the sensory integrative ability for postural control when vision was deprived, and the somatosensory input was unreliable compared to the control group.

The findings of this study suggest that vestibular-focused balance training may help to reduce the frequency of falls in patients with mild and moderate PD. The results are consistent with a high-quality study that employs a 6-month duration, predominantly home-based, minimally supervised exercise regimen focusing on mitigating fall risk factors; the results indicated a decrease in falls among people with mild PD, while no significant reduction was observed among those with more severe PD [18]. Similarly, in a separate study, a secondary analysis revealed that participants with moderate disease severity experienced decreased fall rates within the experimental group, while individuals with severe disease did not exhibit significant reductions in falls [25].

The structured nature of the study group intervention likely contributed to its effectiveness by providing participants with targeted exercises and support to improve motor function and reduce the risk of falls.

The data indicated that both groups showed a significant improvement in FGA scores. The improvement did not last till Week 12. Furthermore, the changes observed in the control group did not exceed the minimal detected change (MDC) for the FGA in the PD population [26]. The difference in training effect could have been the result of the specificity of the training situation offered by vestibular-oriented balance training to increase vestibular feedback. Although people with PD have been found to experience impaired motor learning, previous research has indicated that they may still retain the ability to learn new tasks [27].

The observation of increased activity in the cerebellum of patients with PD while performing motor tasks suggests that the cerebellum might play a role in the acquisition of motor skills. This could make subsequent motor learning processes independent of the striatum and unaffected by their dopamine levels. Another potential candidate for the compensatory motor learning process is the hippocampus. Similarly to the cerebellum, the hippocampus tends to remain relatively unaffected in the early stages of PD, as seen in the patients included in the study conducted by Hawkes et al. [28]. Thus, it is possible that the hippocampus could be recruited to support motor learning. The results are consistent with those of Lahlou et al. [29] who investigated whether PD patients have impaired memory of motor learning over time. The author found that motor memory maintenance across a 2-day delay was not impaired in patients compared to older controls and was not influenced by the dopamine state at the time of initial learning. Specifically, performance was maintained over a 2-day delay in patients to the same extent as in older controls.

Neurophysiological studies have suggested that the cerebellum is responsible for supervised and feedforward learning, while the basal ganglia play a role in providing internal feedback to optimize rewards [27, 30]. Furthermore, the cerebellum has been identified as a crucial neural module for integrating various sensory inputs, such as visual, vestibular, and somatosensory information, to execute the vestibular spinal reflex, which aids in postural control. Considering these findings, it is possible that the individuals with impaired basal ganglia in our study might compensate by relying more efficiently on the cerebellum to integrate visual and vestibular information. This, in turn, could influence the brain stem and spinal cord, leading to improvements in postural control.

The findings of this study are in line with those of Freidle et al. [31], who observed a slight impairment in implicit motor sequence learning among individuals with PD compared to healthy individuals. In particular, this impairment did not show statistically significant group differences in the task-related functional magnetic resonance imaging (fMRI) data. A plausible interpretation of these results is that people with PD require more time to achieve a level of implicit motor sequence learning similar to that of their healthy counterparts. In this study, a strong emphasis on a high frequency of exercise repetitions was utilized, both during therapy sessions and at home, ranging from as many as 288 repetitions per week for each exercise. Additionally, the treatment protocol is extended to 8 weeks to enhance motor learning and facilitate retention. This observed improvement could potentially be attributed to the impact of high-intensity exercise on modulating cortical excitability in patients with PD. This modulation could arise from the increased quantity (repetitions) and consistency of exercises, all geared toward reinforcing vestibular inputs. It is worth noting that these effects are not fleeting but could be sustained 4 weeks after the conclusion of the training [32]. Lastly, based on the results of animal studies, high-intensity exercises may facilitate the release of neurotrophic factors believed to underlie improved motor function [33, 34].

In a study comparing vestibular-focused balance training with a control group, the trained group showed a significant improvement in sensory integrative ability for postural control, especially when vision was deprived, and somatosensory input was unreliable. Both groups showed improvements in stable surface conditions with eyes open and closed, indicating improved sensory integration. However, the study group showed a significantly greater mean difference in these conditions compared to the control group. Furthermore, the study group demonstrated improvements in conditions that included a compliant surface and altered visual input, relying more on the vestibular system for postural adjustments, while the control group did not show significant changes. Although the study group experienced a decrease in scores between Weeks 8 and 12, they retained the overall improvement compared to baseline measurements. This was supported by a significant improvement in performance from baseline assessment to Week 12. The absence of a significant difference between the groups in Conditions 1 and 2 of the mCTSIB may be attributed to the application of comparable traditional balance training principles to both groups, including monitoring visual and somatosensory cues during training. The crucial distinction between the training groups is that the study group exhibited significant improvements in Conditions 3 and 4 (involving unreliable somatosensory and visual conditions). This disparity in training outcomes could be attributed to the training scenario in the vestibular focus group that mirrors the testing conditions of mCTSIB-3 and 4. Vestibular-focused training introduced activities that facilitated feedback from the compliant surface through self-initiated weight shifts (internal feedback). This context likely contributed to the enhanced central processing and integration of vestibular information, particularly in situations characterized by unreliable somatosensation and vision. The findings align with those of Frenklach and colleagues [35], who observed that people with PD struggled to use impaired proprioceptive feedback on dynamic moving surfaces for orientation. Consequently, they relied more heavily on vestibular and visual feedback, or exclusively on vestibular feedback. This resonates with the notion that people with PD do not rely heavily on visual input. Therefore, the integrity of vestibular function emerges as crucial for maintaining postural stability in this population [36].

This interpretation also agrees with earlier studies suggesting that while the cerebellum is responsible for feedforward and guided learning, the basal ganglia play a significant role in providing internal feedback to optimize rewards [37, 38]. Additionally, the cerebellum emerges as a critical neural hub for integrating various sensory inputs, visual, vestibular, and somatosensory, enabling the execution of vestibular spinal reflexes that aid in postural control [39]. Therefore, individuals with impaired basal ganglia function, as in this study, could exhibit enhanced integration of visual and vestibular information, facilitated through the cerebellum. This, in turn, could influence brain stem and spinal cord function, ultimately improving postural control.

In particular, the results of this study revealed a marginal significant reduction in the incidence of falls among the study group, which retained substantial improvements in both Condition 4 of mCTSIB and FGA scores. This finding aligns with previous research indicating that instability during the last sensory-conflicting condition test of the sensory organization, analogous to Condition 4 of mCTSIB, accurately predicted the risk of falling [40]. Additionally, the FGA demonstrated strong construct validity in patients with PD, effectively predicting falls in the subsequent 6 months [19]. Therefore, vestibular-focused balance training holds promise for preventing falls, particularly under conditions of compromised visual and somatosensory inputs.

### 4.1. Limitations

The primary limitation of this study is related to the method used to record falls. Specifically, participants did not receive a logbook to document fall events immediately after their occurrence. Instead, the study relied on the participants' recollection of fall incidents during the follow-up period. This reliance on memory recall introduces the potential for inaccuracy, as individuals or their caregivers may not recall fall events with precision due to a variety of factors. Furthermore, there is a likelihood of bias toward remembering more recent or severe falls, skewing the overall accuracy of fall history recall.

Given the progressive degenerative nature of PD, it is important to acknowledge that the conclusions drawn from this study, based on a 1-month follow-up period, should not be extrapolated to represent a patient's long-term fall risk. The dynamic nature of PD suggests that the risk of falling can evolve over long periods.

To address this limitation, future research efforts could consider extending the duration of follow-up to gain insight into the prediction of long-term fall risk, as indicated by the FGA. This approach would provide a more comprehensive understanding of how the risk of falls evolves in the context of PD and would provide valuable information for patient management and care.

## 5. Conclusion

The study suggests that vestibular-oriented balance training could be an effective intervention to improve postural control and decrease the risk of falls in individuals with mild-to-moderate PD. This would suggest that physical therapists may consider intervening earlier in the disease process with balance interventions intended to reduce falls. The findings provide valuable information for healthcare professionals and clinicians involved in the rehabilitation and treatment of patients with PD, forming the development of evidence-based interventions and rehabilitation programs.

## Figures and Tables

**Table 1:** Mean (SD) of baseline characteristics by study group (*N* = 40).

	Study (*n* = 20)	Control (*n* = 20)	*p* value
Male (*n*)	17	17	1.00 (0.67)
Age (year)	77.8 (4.6)	76.7 (4.6)	0.45
Height (m)	1.7 (0.05)	1.7 (0.05)	0.60
Weight (kg)	78.9 (5.9)	78.5 (6.5)	0.86
BMI (kg/m^2^)	26.7 (1.5)	26.9 (1.8)	0.77
HTN (*n*)	15	15	1.00 (0.64)
DM (*n*)	7	7	1.00 (0.63)
Cardiac dysfunction (*n*)	13	12	1.00 (0.50)
Falls (*n*, %)	15 (75)	14 (70)	1.00 (0.50)
Fall frequency, median (min, max)	1 (0, 2)	1 (0, 2)	0.86

Abbreviations: BMI, body mass index; kg, kilogram; m, meter; SD, standard deviation.

**Table 2:** Number (%) of falls by study group (*N* = 40).

	Study group (*n* = 20)	Control group (*n* = 20)
Baseline	15 (75)	14 (70)
8 weeks	9 (45)⁣^∗^	13 (65)
12 weeks	11 (55)	13 (65)

⁣^∗^A marginal significant drop in the number of falls by Week 8 (*p*=0.038 without Yates; *p*=0.09 with yates).

**Table 3:** Median (minimum, maximum) of frequency of falls by study group (*N* = 20).

	Study group (*n* = 15)	Control group (*n* = 14)	*p* value (*r*)
Baseline	1 (0, 2)	1 (0, 2)	0.86 (0.03)⁣^∗^
8 weeks	0 (0, 1)	1 (0, 2)	0.11 (0.126)⁣^∗^
12 weeks	0 (0, 1)	1 (0, 2)	0.27 (0.18)⁣^∗^

⁣^∗^Mann–Whitney *U*-test.

**Table 4:** Mean (SD) of outcome variables by study group over time (*N* = 40).

	Study (*n* = 20)			Control (*n* = 20)			*p* value over time (*η*^2^)⁣^∗∗^	p value (group × time) (*η*^2^)⁣^∗∗^
	Baseline	8 weeks	12 weeks	Baseline	8 weeks	12 weeks		
FGA	15.50 (1.91)	22.15 (1.46)⁣^∗^	20.70 (1.53)⁣^∗^	14.95 (2.42)	16.90 (2.88)⁣^∗^	16.55 (2.61)	< 0.001 (0.68)	< 0.001 (0.38)
mCTSIB SSEO	1.37 (0.34)	0.92 (0.16)⁣^∗^	0.99 (0.17)⁣^∗^	1.29 (0.38)	1.14 (0.24)⁣^∗^	1.18 (0.23)	< 0.001 (0.49)	< 0.001 (0.21)
mCTSIB SSEC	3.42 (0.91)	1.91 (0.52)⁣^∗^	1.98 (0.57)⁣^∗^	3.67 (0.91)	2.82 (0.72)⁣^∗^	3.05 (0.63)	< 0.001 (0.78)	< 0.001 (0.29)
mCTSIB CSEO	2.13 (0.44)	1.06 (0.24)⁣^∗^	1.24 (0.27)⁣^∗^	2.35 (0.88)	2.23 (0.82)	2.29 (0.93)	< 0.001 (0.68)	< 0.001 (0.58)
mCTSIB CSEC	5.45 (1.64)	2.97 (0.93)⁣^∗^	3.20 (0.94)⁣^∗^	5.60 (1.67)	5.22 (1.38)⁣^∗^	5.41 (1.60)⁣^∗^	< 0.001 (0.71)	< 0.001 (0.60)

*Note:η* ^2^, partial eta squared.

Abbreviations: CSEO, compliant surface eyes closed; CSEO, compliant surface eyes open; mCTSIB, modified Clinical Test of Sensory Interaction in Balance; SD, standard deviation; SSEC, stable surface eyes closed; SSEO, stable surface eyes open.

⁣^∗^One-way repeated measures ANOVA, ≤ 0.05.

⁣^∗∗^Mixed factorial ANOVA, *p* ≤ 0.05.

**Table 5:** Mean (SD) changes from baseline by study group.

	Time change from baseline	Study (*n* = 20)	Control (*n* = 20)	Mean difference (95% CI)	*p* value (Cohen's *d*)⁣^∗^
FGA	Week 8	6.65 (1.39)	1.95 (2.95)	4.70 (3.23, 6.17)	< 0.001 (2.04)
	Week 12	5.20 (1.44)	1.60 (2.95)	3.60 (2.12, 5.08)	< 0.001 (1.55)

mCTSIB SSEO	Week 8	−0.45 (0.29)	−0.15 (0.27)	0.30 (0.12, 0.48)	0.002 (1.08)
	Week 12	−0.38 (0.24)	−0.11 (0.27)	0.28 (0.11, 0.44)	0.001 (1.06)

mCTSIB SSEC	Week 8	−1.51 (0.55)	−0.85 (0.49)	0.66 (0.32, 0.99)	0.001 (1.25)
	Week 12	−1.44 (0.68)	−0.62 (0.51)	0.83 (0.43, 1.21)	0.001 (1.38)

mCTSIB CSEO	Week 8	1.07 (0.35)	−0.12 (0.28)	0.95 (0.75, 1.16)	0.001 (2.98)
	Week 12	−0.89 (0.35)	0.07 (0.30)	0.82 (0.61, 1.03)	0.001 (2.51)

mCTSIB CSEC	Week 8	−2.48 (0.97)	−0.38 (0.60)	2.10 (1.58, 2.61)	0.001 (2.61)
	Week 12	−2.26 (1.09)	0.19 (0.30)	2.07 (1.56, 2.58)	0.001 (2.59)

Abbreviations: CSEO, compliant surface eyes closed; CSEO, compliant surface eyes open; mCTSIB, modified Clinical Test of Sensory Interaction in Balance; SD, standard deviation; SSEC, stable surface eyes closed; SSEO, stable surface eyes open.

⁣^∗^Independent *t*-test, *p* ≤ 0.05.

## Data Availability

The data that support the findings of this study are available on request from the corresponding author. The data are not publicly available due to privacy or ethical restrictions.
